# Tracing the origin of the early wet‐season *Anopheles coluzzii* in the Sahel

**DOI:** 10.1111/eva.12486

**Published:** 2017-05-26

**Authors:** Tovi Lehmann, David Weetman, Diana L. Huestis, Alpha S. Yaro, Yaya Kassogue, Moussa Diallo, Martin J. Donnelly, Adama Dao

**Affiliations:** ^1^ Laboratory of Malaria and Vector Research NIAID, NIH Rockville MD USA; ^2^ Department of Vector Biology Liverpool School of Tropical Medicine Liverpool UK; ^3^ Malaria Research and Training Center (MRTC) Faculty of Medicine, Pharmacy and Odonto‐stomatology Bamako Mali

**Keywords:** aestivation, dormancy, effective population size, malaria, migration, Sahel, seasonality, single‐nucleotide polymorphisms, vector‐borne diseases

## Abstract

In arid environments, the source of the malaria mosquito populations that re‐establish soon after first rains remains a puzzle and alternative explanations have been proposed. Using genetic data, we evaluated whether the early rainy season (RS) population of *Anopheles coluzzii* is descended from the preceding late RS generation at the same locality, consistent with dry season (DS) dormancy (aestivation), or from migrants from distant locations. Distinct predictions derived from these two hypotheses were assessed, based on variation in 738 SNPs in eleven *A. coluzzii* samples, including seven samples spanning 2 years in a Sahelian village. As predicted by the “local origin under aestivation hypothesis,” temporal samples from the late RS and those collected after the first rain of the following RS were clustered together, while larger genetic distances were found among samples spanning the RS. Likewise, multilocus genotype composition of samples from the end of the RS was similar across samples until the following RS, unlike samples that spanned the RS. Consistent with reproductive arrest during the DS, no genetic drift was detected between samples taken over that period, despite encompassing extreme population minima, whereas it was detected between samples spanning the RS. Accordingly, the variance in allele frequency increased with time over the RS, but not over the DS. However, not all the results agreed with aestivation. Large genetic distances separated samples taken a year apart, and during the first year, within‐sample genetic diversity declined and increased back during the late RS, suggesting a bottleneck followed by migration. The decline of genetic diversity followed by a mass distribution of insecticide‐treated nets was accompanied by a reduced mosquito density and a rise in the mutation conferring resistance to pyrethroids, indicating a bottleneck due to insecticidal selection. Overall, our results support aestivation in *A. coluzzii* during the DS that is accompanied by long‐distance migration in the late RS.

## 1 INTRODUCTION

Malaria in Africa extends from the humid equatorial forest to semi‐arid zones in the north and south. Three species within the *Anopheles gambiae* species complex, *A. gambiae, A. coluzzii* and *A. arabiensis* are primary vectors of malaria in Africa. Beyond the equatorial zone, malaria transmission is seasonal and in regions where surface waters required for larval development are absent during the 3‐ to 7‐month‐long dry season (DS), mosquito densities drop dramatically (Adamou et al., 2011; Dao et al., 2014; Lehmann et al., 2010; Lemasson et al., 1997; Omer & Cloudsley‐Thompson, 1968, 1970; Simard, Lehmann, Lemasson, Diatta, & Fontenille, 2000). Whether populations survive the long DS by aestivation (a “semi‐dormant” state promoting extended longevity during the DS) or are re‐established by migrants from distant locations where larval sites are available year‐round has remained an enigma for over 60 years (Donnelly, Simard, & Lehmann, 2002; Gillies & De Meillon, 1968). Continued interest in this question is fuelled by recognition that the fragile DS populations are an ideal target for malaria control. Recent studies have amassed evidence that in the West African Sahel *A. coluzzii* aestivates, whereas *A. gambiae* re‐establishes populations via long‐distance migration (Adamou et al., 2011; Arcaz et al., 2016; Dao et al., 2014; Huestis et al., 2012; Lehmann et al., 2010; Mamai, Mouline, et al., 2016; Mamai, Simard, et al., 2016; Yaro et al., 2012). However, direct evidence such as observation of aestivating adults in their hidden shelters or the recapture of marked mosquitoes hundreds of kilometres from their release sites remains elusive; thus, additional indirect evidence could help understand this problem. Here, we undertake a population genetic study to evaluate and distinguish between these explanations of persistence throughout the DS.

Previous genetic studies have aimed to detect seasonal bottlenecks in different populations of the *A. gambiae* species complex that undergo severe density depression during the DS or that have undergone vector control (Athrey et al., 2012; Hodges et al., 2013; Lehmann, Hawley, Grebert, & Collins, 1998; Simard et al., 2000; Taylor, Toure, Coluzzi, & Petrarca, 1993; Wondji, Simard, et al., 2005). The effective population size (*N*_e_) approximates the number of breeding adults in a population if the population density is stable and parental contributions to the next generation are randomly distributed (Nei & Tajima, 1981; Waples, 1989). However, over a series of generations, it will be most affected by the lowest *N*_e_ in any generation, as the harmonic mean. Studies of *A. gambiae* have revealed that except for in cases of strong vector control, *N*_e_ was large (*N*_e_ ≥ 1,000) and commonly the upper 95% CI included infinity, consistent with a robust aestivating population or with mass migration. This also indicates that the statistical power derived from genotyping specimens at up to 12 microsatellite loci with ~90 alleles is insufficient for the estimation of *N*_e_ of these populations. Shallow genetic structure typically characterizes populations of *A. gambiae* and *A. coluzzii* (excluding differences between inverted and standard chromosomal inversions) over large distances (Czeher et al., 2010; Lehmann et al., 1996, 2003; Pinto et al., 2013; Slotman et al., 2007; Weetman, Wilding, Steen, Pinto, & Donnelly, 2012), supporting the presence of large *N*_e_, as well as a formidable migration force (gene flow).

Our goal was to determine whether the early rainy season (RS) population of *A. coluzzii* in the Sahel descend from the preceding late RS generation from the same locality, consistent with aestivation (hypothesis 1), rather than from distinct and presumably distant locations, consistent with migration (hypothesis 2). The former hypothesis was proposed for *A. coluzzii* and the latter for *A. gambiae,* respectively (Dao et al., 2014). The expectation of a DS population bottleneck typically defined as *N*_e_ < 100 (Avise, 1994; Luikart, Allendorf, Cornuet, & Sherwin, 1998) would reflect that the few mosquitoes observed in the DS are survivors that persist until the following rains. However, reduced numbers but no bottleneck is expected if hundreds or even thousands of, mostly unobserved, mosquitoes successfully undergo aestivation as suggested by recent studies (Adamou et al., 2011; Dao et al., 2014). Alternatively, local Sahelian populations may go extinct during the DS and become re‐established at the early RS by migrants from areas with permanent water facilitating continued breeding. These hypotheses lead to distinct predictions in terms of the within‐population genetic diversity and differentiation between populations as summarized in Table 1.

**Table 1:** Key predictions based on aestivation and long‐distance migration hypotheses

Parameter	Aestivation	Long‐distance migration
Within‐sample genetic diversity	(1.1) high and stable within‐population diversity is expected over time between samples taken at the same Sahelian location, notably (1.2) when comparing the late DS (or shortly after the first rain) with the late RS.	(2.1) within‐population diversity would be low and unstable across time, notably (2.2) when comparing the late DS (or shortly after the first rain) with late RS, owing to a founder effect.
Between sample genetic distance	(1.3) clustering of temporal samples based on genetic distance is expected to group together samples taken across seasons in one Sahelian site separately from other localities, reflecting ancestral continuity and survival of many aestivators throughout the DS.	(2.3) clustering of temporal samples based on genetic distance is expected to separate samples taken across dry and rainy seasons in one Sahelian locality, reflecting founder effects at the early RS by migrants arriving from distant locations
Temporal variance in allele frequency	(1.4) owing to reproductive arrest during the Sahelian DS, negligible genetic drift is expected between samples taken at the same locality during this time frame.	(2.4) large genetic drift is expected between samples taken at the same location between the DS or early RS and the late RS.

**Figure 1:**
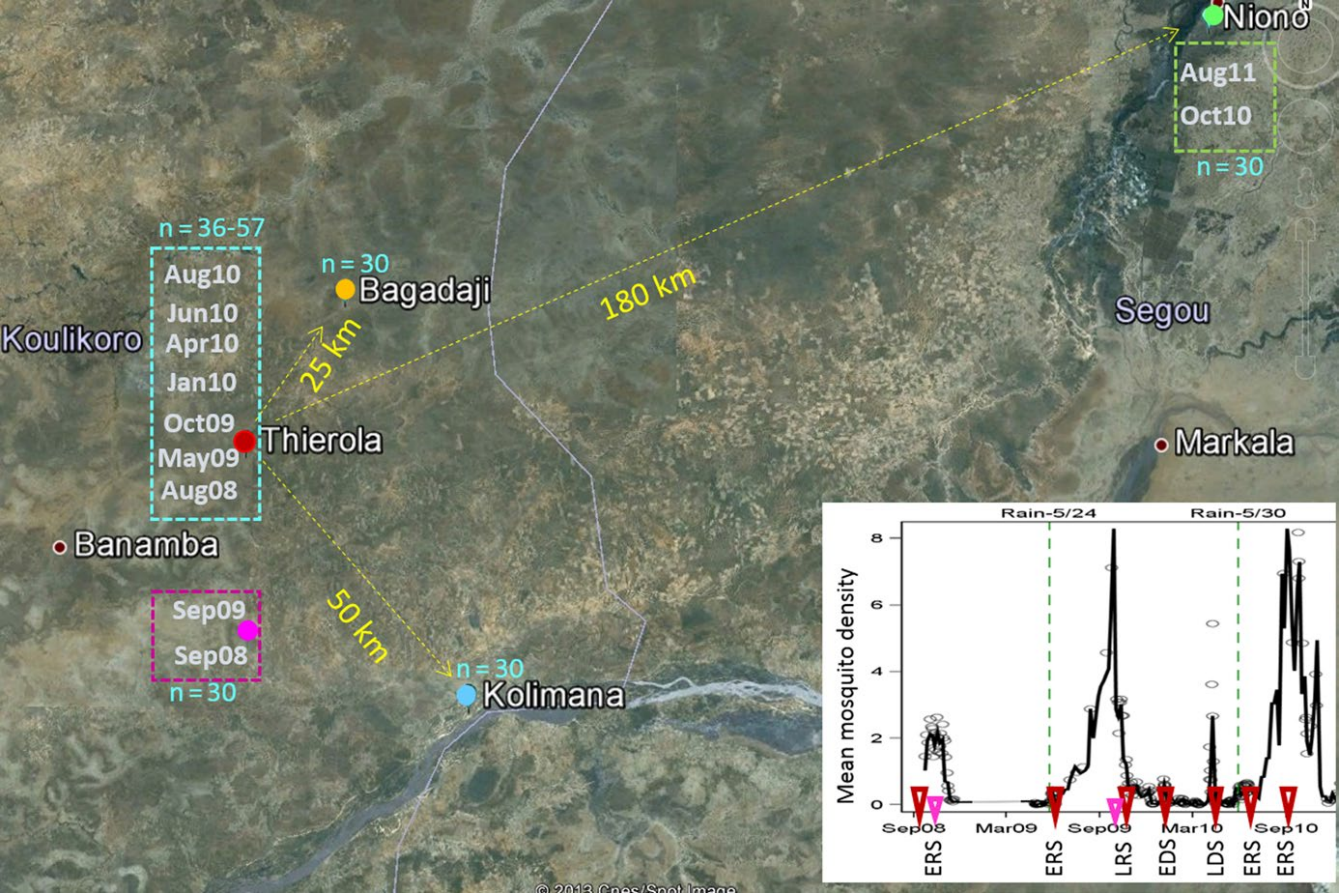
Map of study populations (coloured dots) with samples from Thierola named after the month and year of collection (*n* = sample size). Distances from Thierola are shown (yellow). Inset: Temporal sequence of sample collection dates from Thierola (red and purple for *Anopheles coluzzii* superimposed on population density (mean *Anopheles gambiae s.l*./house, Dao et al., 2014). Observed daily mean densities (in ~120 houses) are shown as circles and mean density over 5 days intervals is shown by the line. The seasonal acronyms under the *x*‐axis includes “RS” and “DS” to denote rainy and dry seasons, preceded by “E” or “L” to denote the early and late parts of each season, respectively

By including eleven samples of *A. coluzzii* and two of *A. gambiae* in this study, we focus the tests on the former species with only exploratory analysis of the second. In total, we genotyped 520 mosquitoes at 1,536 single‐nucleotide polymorphisms (SNPs). The specimens represent seven time points of *A. coluzzii* spanning 2 years from our focal Sahelian village, Thierola, and an additional four samples from three neighbouring villages, as well as two samples of *A. gambiae* from Thierola spanning 1 year (Figure 1, Table 2). Of the four collection sites, Thierola and Bagadaji offer no surface water for larval sites for 4–6 months a year during the DS, whereas larval sites are available year‐round in Niono and Kolimana. By describing patterns of genetic variation within and between species, populations and samples, and testing the predictions in Table 1, we evaluate whether the ecological strategies used by *Anopheles* malaria vectors to persist in the Sahel fit with current hypotheses.

**Table 2:**
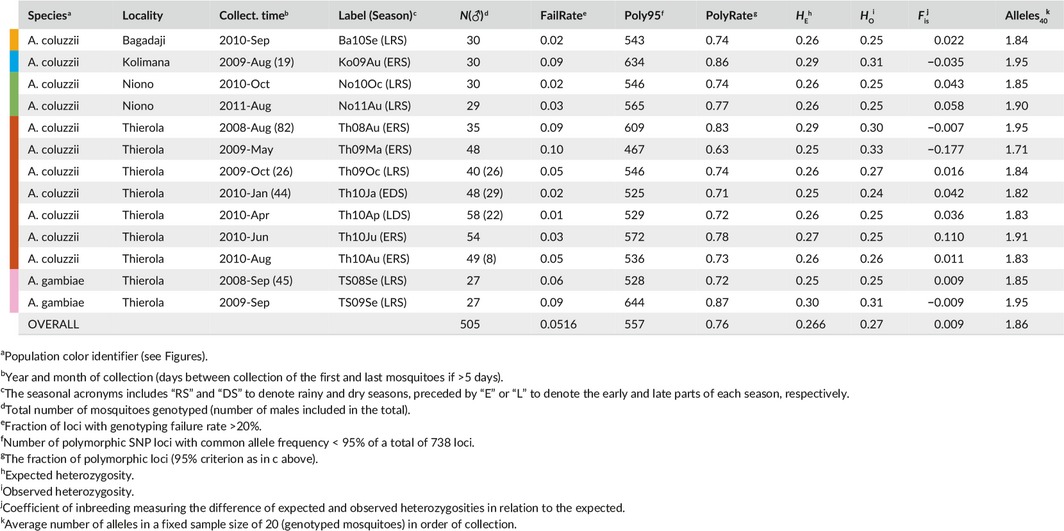
Sample size, polymorphism and genetic diversity in 738 SNP loci

## 2 MATERIALS AND METHODS

### 2.1 Mosquito collection

Live indoor‐resting mosquitoes were collected in the morning from houses by aspiration in Thierola [13.6586°N, 7.2147°W] and Niono (Soukrani village [14.2205° N, 6.0499°W]) and by pyrethrum spray collection in Bagadaji [13.8426°N, 7.0954°W] and Kolimana [13.3536°N, 6.9309°W] (Adamou et al., 2011; Lehmann et al., 2010). Seven temporally spaced samples of *A. coluzzii* were obtained from the Sahelian village Thierola, two from Niono and a single sample from each of the villages of Kolimana and Bagadaji (Table 2 and Figure 1). Two temporally spaced samples of *A. gambiae* were obtained from Thierola (Table 2 and Figure 1). Hereafter, population refers to locality (and species), whereas sample refers to a particular collection time point for a given population. During the DS, there is no surface water nor anopheline breeding sites in the villages of Thierola and Bagadaji and mosquito density is very low (Adamou et al., 2011; Dao et al., 2014; Lehmann et al., 2010). The village of Kolimana sits along the bank of the Niger River (50 km East of Thierola), and there are at least a few larval sites available year‐round. The village of Soukrani (Niono) is surrounded by a large rice irrigation system where the year‐round availability of many larval sites support an extremely high density of *A. coluzzii* (Diuk‐Wasser et al., 2007; Sogoba et al., 2007). Samples were obtained during the RS (July to October), the DS (December to May) and during the transition months (June and November; Figure 1). Most samples were collected over a maximum of 5 days, but several such as *A. coluzzii* from Thierola in August 2008 were collected over a longer period (Table 2).

### 2.2 DNA extraction and genotyping

Following morphological identification (Gillies & De Meillon, 1968), only *A. gambiae* complex mosquitoes were included, with identity refined using sequential species‐specific PCR and PCR‐RFLP with legs as template (Fanello, Santolamazza, & della Torre, 2002). DNA from individual mosquitoes was extracted using the Qiagen DNEasy kit and quantified using the PicoGreen fluorimetric assay (Invitrogen). As DNA extracts from individual mosquitoes typically contain insufficient DNA for high‐throughput genotyping assays (Wilding, Weetman, Steen, & Donnelly, 2009), whole genome amplification was required using 50 ng of DNA extract as template for the GenomiPhi V2 DNA Amplification Kit (GE Life Sciences). Following whole genome amplification, each sample was quantified using PicoGreen and diluted to yield 50 ng/μl, 5 μl served as template for an Illumina GoldenGate SNP assay, which we ran on an Illumina Beadstation GX according to the manufacturer's protocols. Specimens were genotyped used a custom 1536‐SNP array, enriched for insecticide resistance candidate genes with ≈20% of the SNPs located in control, intergenic regions or noncoding regions distributed through the genome (Weetman et al., 2010). The version of the array applied here replaced SNPs which consistently failed in an earlier version, and provided more balanced genomic coverage (Weetman et al., 2014). Genotype calls were made with Beadstudio v3.2 (Illumina Inc.) with all calls checked manually (Weetman et al., 2010, 2014).

### 2.3 Data analysis

Quality of SNP scores was carefully evaluated and 619 SNPs were excluded because of poor scoring or a low genotype conversion rate across mosquitoes (<80%). To simplify concurrent analysis of males and females, the relatively few X‐linked loci (*N* = 65) were removed from the analysis. Following evaluation of *A. coluzzii* samples only, a further 40 SNPs were excluded because of extreme deviation from Hardy–Weinberg proportions (using a strict Bonferroni‐corrected threshold of α = 0.05/*N* SNPs), and a further 74 because of extremely low polymorphism (major allele frequency, MAF > 99%). This yielded a final set of 738 SNPs which were included in the present analysis. Similarly 14 mosquitoes (spread across samples: Kolimana [4], and the Thierola samples from August 2008, May 2009, October 2009 and January 2010 [3, 1, 4 and 2, respectively]), with over 10% genotyping failure rate were also removed, leaving a total sample size of 510 mosquitoes.

Within‐sample genetic diversity was measured as the number of polymorphic SNPs (major allele frequency <95%); expected heterozygosity (*H*_e_) per locus; and mean number of alleles per locus. To accommodate variation in sample size, these statistics were also computed based on the smallest sample size (*n* = 40 chromosomes), selecting the first 20 mosquitoes in larger samples. The differences between samples (*n* = 40 *A. coluzzii*) in the mean heterozygosity and the mean number of alleles were evaluated using ANOVA, treating locus as a random variable and sample as a fixed parameter and using contrasts to estimate differences between samples according to *a priori* hypotheses (see predictions in Table 1). The analysis used Proc Mixed (SAS 9.4). Exact goodness‐of‐fit tests were used to assess deviations of genotype composition from Hardy–Weinberg expectations (HWE) in each locus and sample, accommodating multiple tests as discussed below. The inbreeding coefficient, Fis, measuring the difference of observed heterozygosity from *H*_e_ in units of *H*_e_ (Nei, 1987; Wright, 1951) were used to describe such deviations.

The Euclidian distance (SQRT(sum[MAF_i _− MAF_j_]^2^), where MAF denotes major allele frequency in the pooled *A. coluzzii* sample, while the subscripts i and j denote different samples) across loci was estimated as distance between samples. Unlike Fst, this genetic distance remains meaningful not only in comparisons between populations but also between samples representing different time points of the same population. The distance matrix among all sample pairs computed by Proc Distance (SAS) was used to generate a dendrogram using an UPGMA tree building algorithms as well as 2D plots produced by subjecting the distance matrix to multidimensional scaling analysis (MDS). Neighbour joining population trees were plotted using MEGA 4 (Tamura, Dudley, Nei, & Kumar, 2007) based on the distance matrices described above.

To identify subgroups without prior information on species, geographical location or time point, we used STRUCTURE 2.3.3 (Pritchard et al. 2000). An admixture model assuming correlated allele frequencies was used throughout. Preliminary runs with the possible number of subpopulations (clusters, *K*) set between 1 and 10 employed 20,000 iterations, the first 10,000 as burn‐in. Probability plots suggested a maximum of *K* = 6 or 7, and also a rapid plateau in parameter values within the burn‐in period. Consequently, these were followed by test runs of 50,000 iterations, again with a burn‐in of 10,000, with *K* set between 1 and 7. Each value of *K* was replicated five times, very low variability in log probability values detected among runs following processing suggesting that additional replicates would not alter the optimum solution. Results were processed with Structure Harvester v0.6.94 (Earl & Vonholdt, 2012). The optimal value was determined using the deltaK method (Evanno, Regnaut, & Goudet, 2005). Subpopulation membership of individuals was determined by the highest ancestry proportion (Q) of each individual.

To test whether genetic drift between samples collected during the DS (and immediately after the first rains) has occurred, we partitioned the temporal variation in allele frequency (*F* ^*T*^, Krimbas and Tsakas 1971; Nei & Tajima, 1981; Pollak 1983) to its components: the experimental sampling variance and the genetic variance (Ne). Thus, if *F* ^*T*^ was larger than the experimental sampling variance, genetic drift was measurable, consistent with reproduction over one or more generations. On the other hand, if *F* ^*T*^ was smaller than (or equal to) the experimenter's sampling variance, genetic drift was not evident, consistent with no reproduction. The genetic component is more difficult to detect the larger Ne is (Nei & Tajima, 1981; Waples, 1989). Importantly, during the DS and immediately after the rains, density and thus Ne are thought to be at their lowest; hence, genetic drift should be evident if reproduction takes place. Under reproductive arrest (aestivation), no drift can be measured because no sampling of gametes took place, according to our prediction 1.4 (Table 1). Instead, drift should be detected during the RS, when density and thus Ne are large yet reproduction is ongoing, facilitating the biological sampling of gametes between generations. While it might appear theoretically easier to detect drift during the DS, when density is the lowest, it should be impossible if there is no reproduction. On the other hand, under migration, temporal samples reflect reproductive cycles either at the origin or *in situ*, as well as founder effects, further increasing drift. For each pair of samples, *F* ^*T*^ and the experimental sampling variance (*V* ^*T*^) were computed according to Nei and Tajima (1981):

*F* ^*T*^ = (*x*_*i *_− *y*_*i*_)^2^/[(*x*_*i*_ **+ *y*_*i*_)/2 − (*x*_i_*y_*i*_)] and *V* ^*T*^ = (1/*S*_*xi*_) + (1/*S*_*yi*_), where *x*_*i*_ and *y*_*i*_ are the common allele in locus *i* (all di‐allelic) in the first (*x*) and second (*y*) samples, and *S*_*xi*_ and *S*_*yi*_ are the sample sizes (number of chromosomes) of the first (*x*) and second (*y*) samples from that locus (*i*). The Wilcoxon rank test was used to test whether the median of the distribution of the difference (*F* ^*T*^_*i*_ − *V* ^*T*^_*i*_) over all loci was equal or larger than zero to determine whether it *F* ^*T*^ > *V* ^*T*^ for each pair of samples.

Global tests were employed to evaluate significance of multiple tests. The sequential Bonferroni procedure (Holm, 1979) was applied to detect a single test‐specific departure (e.g., for a locus), whereas the binomial test (which estimates the probability of obtaining the observed number of significant tests at a given significance level given the total number of tests) can detect weaker departures across multiple tests, such as genomewide departures. Unless otherwise specified, calculations were carried out using programmes written by TL in SAS (2011).

## 3 RESULTS

Excluding invariant positions across all samples and applying the stringency criteria explained above (see Section 2), a total of 738 loci were selected for the analysis. These consisted of 31 replacement (4.2%), 126 synonymous (17.1%) and 581 substitutions in noncoding regions (78.7%, Fig. S1). A total of 95 SNPs (12.9%) were located in polymorphic inversions (58 in 2La and 37 in 2Rbc, Fig. S1). Genotyping success rate averaged 95% across samples (range 90%–99%, Table 2), possibly reflecting variation in the quality of DNA preservation.

### 3.1 Within‐sample genetic diversity

**Figure 2:**
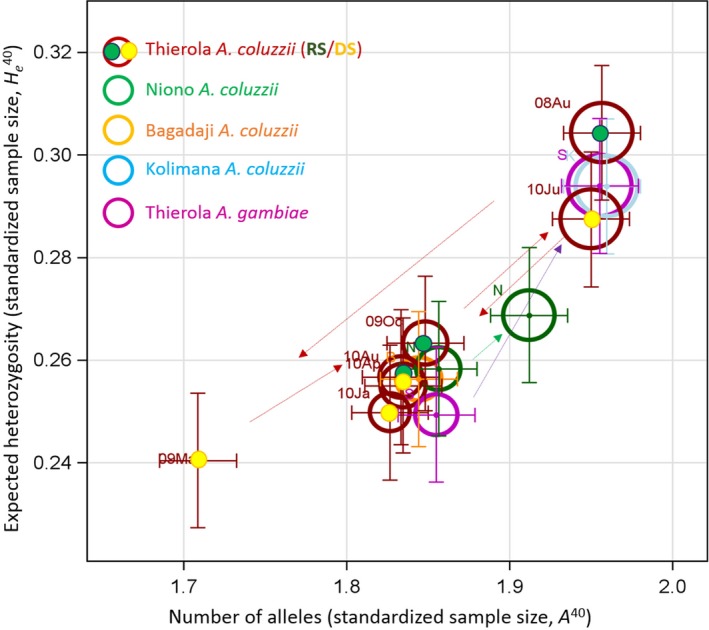
Within‐sample genetic diversity (mean and 95% CI) in a standardized sample size (*N* = 20 individuals, see text). Bubble size is proportional to the fraction of polymorphic loci (major allele frequency < 95%). Least‐squares means and 95% CI of expected heterozygosity (*H*_e_ *)* on the *y*‐axis and the number of alleles (*A*) on the *x*‐axis, computed in an ANCOVA, with locus treated as a random effect and population and the frequency of the common allele as fixed effects, are shown by lines (sample sizes are given in Table 2). Significant change in diversity (both *H*_e_ and *A*) based on nonoverlapping 95% CI of samples in chronological order are marked by dotted arrows (point towards the present). See text for the sample composition of each cluster

The number of polymorphic loci, *P*_95_ (major allele frequency, MAF < 95%), among samples varied considerably (63%–87%) in the full and in the standardized data set, with sample size fixed at *n* = 40 chromosomes (55%–82%, χ^2^_12_ = 206, *p* < .0001, Table 2 and Figure 2). Similarly, expected heterozygosity (*H*_e_) and mean number of alleles/locus (*A*) varied between samples when tested in the standardized data set (*F*_12,8844_ = 32.6, *p* < .0001 for *H*_e_, and *F*_12,8844_ = 52.6, *p* < .0001 for *A*; Figure 2 and Table 2). Although the correlations between these three indices of diversity in the standardized data set were high (*p* < .001, *N* = 13, *r* = 0.91, 0.94 and 0.98 between *H*_e_ *‐A, H*_e_ *‐P*_95_, and *A‐P*_95_, respectively, Figure 2), the variation in *A* revealed deeper separation between samples into three clusters defined by nonoverlapping 95% CI, whereas only two clusters were supported by *H*_e_ (Figure 2). The highest diversity cluster based on *A* comprised of five samples, consisting of *A. coluzzii* from Kolimana, Niono (2011) and Thierola in August 2008 and in June 2010, as well as *A. gambiae* in September 2009. A seven‐sample lower diversity cluster consisted of all the remaining samples, except the lowest, single‐sample cluster of *A. coluzzii* from Thierola in May 2009 (Figure 2). Only two multisample clusters were well separated by *H*_e_, but the rank order of samples remained similar to *A* (Figure 2).

During the first year, diversity in *A. coluzzii* dropped dramatically from the mid‐RS (August 2008) to the time when density surged immediately after the first rain (May 2009) of the following year (Figure 2 and Table 2). The following year, which is represented by five samples, this phenomenon did not repeat: diversity was stable throughout October 2009 to August 2010, including June 2010—the seasonally equivalent sample to May 2009, which actually showed slightly elevated diversity (Figure 2 and Table 2). The inconsistency in stability of within‐population diversity in *A. coluzzii* between years does not fit well with any single prediction. While a drop in diversity in the mid‐RS of the first year to the early rain of the following year might appear to correspond to re‐establishment by migrants, such large changes in genetic diversity have not been observed in previous studies on *A. gambiae* populations, including those from more arid habitats (Lehmann et al., 1998; Simard et al., 2000; Wondji, Simard, et al., 2005), suggesting an exceptional event. A possible explanation for the extreme fluctuation during the first year is a bottleneck associated with the distribution of insecticide‐treated nets in Thierola and surrounding villages in March–May 2008. Changes in the insecticide resistance‐associated mutation *Vgsc*‐1014F, which rose in frequency from 13% to 36% between August 2008 and May 2009 (Fig. S2), could be concordant with such a marked selective impact on local vector populations. An insecticide‐mediated bottleneck might also underlie the low RS density measured in 2008 (Figure 1: Inset). If insecticide effects confounded the first year of the study, the results of the second year would match predictions based on the hypothesis of aestivation rather than on long‐distance migration.

Within‐population diversity (measured by both *A* and *H*_e_, Table 2) was higher in *A. gambiae* than in *A. coluzzii* when tested across all samples and in sympatric samples from the same village (estimated differences of 0.04–0.05 in *A*,* p* < .0001 and 0.5%–0.6% in *H*_e_, *p* < .05, |*t*_contrast_| > 2.0, DF = 1/8,844, Figure 2). In Thierola, *A. gambiae* exhibited the largest range in diversity (*H*_e_ = 0.25–0.30, Table 2) and overall larger variance in *A* and *H*_e_ (*p* < .001, folded *F*_5,165/1,475_ test > 1.2). Populations of *A. coluzzii* in riparian habitats with year‐round access to surface water (Kolimana and Niono) had absolute values of 0.06 higher *A* and 1% higher *H*_e_ (*p* < .001 ANOVA *F*_1/8,844_ > 18, Figure 2) than conspecific populations from habitats with ephemeral water (Bagadaji and Thierola). However, the supposedly very large population of *A. coluzzii* in Niono did not exhibit the coupling of higher genetic diversity with stability that might be expected (Table 2 and Figure 2) with significant variation observed between sampling years (0.056 and 1% in A^40^ and *H*_e_, respectively, *p* < .0001, *F*_1/8,844_ > 27).

Genetic diversity, measured by *H*_e_, was not homogenous across the chromosomes of either species, showing variation between species, populations and temporal samples (Fig. S3). *A. coluzzii* exhibited reduced *H*_e_ on the distal part of 2L and elevated *H*_e_ along the right arm of the 2^nd^ chromosome, whereas *A. gambiae* exhibited higher diversity across the large 2La inversion, but similar or lower diversity near the *Vgsc* locus (Fig. S3A–B). Unlike *A. gambiae*, diversity around the *Vgsc*‐1014F resistance locus was not depressed in *A. coluzzii*. Diversity across the 3^rd^ chromosome was similar for *A. coluzzii* samples, but was heterogeneous in *A. gambiae* (Fig. S3A–B). Diversity of *A. coluzzii* from Kolimana in the left arm of chromosome two was higher than that of Thierola and Niono (Fig. S3C–D). Temporal variation in diversity between Thierolan *A. coluzzii* samples indicated seasonal fluctuation near the 2Rb inversion (Fig. S3E–H).

### 3.2 Within‐sample deviations from HWE

**Figure 3:**
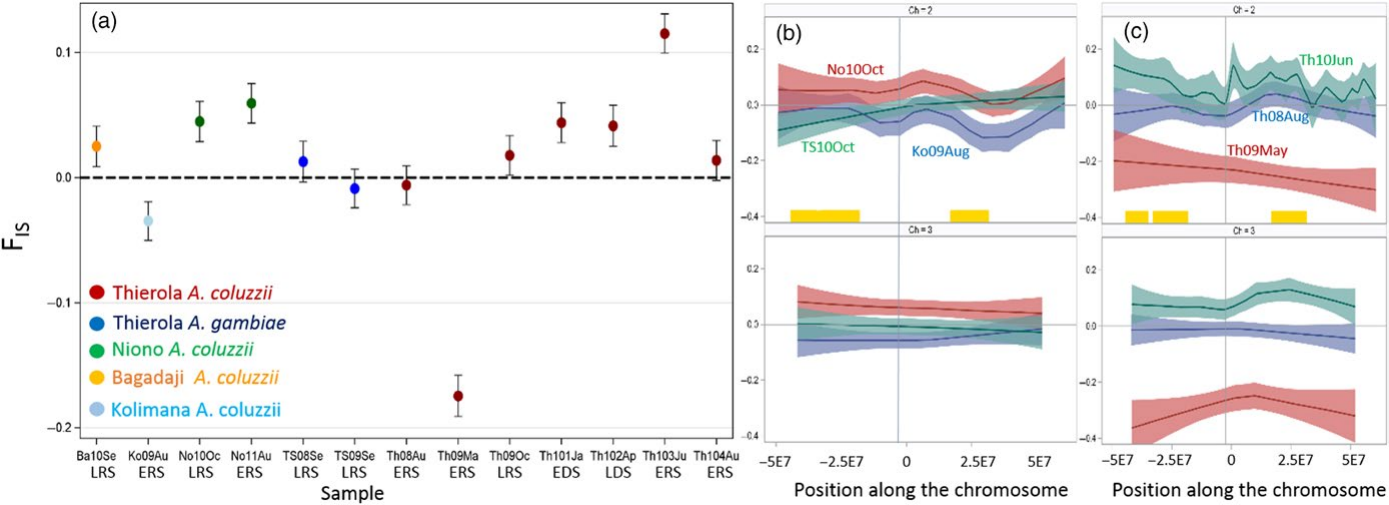
Departures from Hardy–Weinberg equilibrium measured by mean Fis (difference between observed and expected heterozygosity, expressed in units of expected heterozygosity) among samples and across the genome. (a) Least‐squares means of Fis and 95% CI computed in an ANOVA with locus (random effect) and population (fixed effects). Dotted horizontal line marks the expected value (zero). The seasonal acronyms under the *x*‐axis includes “RS” and “DS” to denote rainy and dry seasons, preceded by “E” or “L” to denote the early and late parts of each season, respectively. (b) Variation in Fis along the autosomes (chromosomes 2 and 3 in the top and bottom panels, respectively) in selected samples (note: colours alternate across plots). Nonparametric loess regression functions are used to detect trends and 95% CI (following Fig. S3)

Deviations from HWE showed some extreme Fis values across populations (Table 2 and Figure 3). The mean Fis across all loci and samples was 0.009, but the range of the mean Fis per sample varied widely (−0.17 to 0.11, Table 2). Excluding monomorphic loci (within samples) had little change on the highly significant deviations from HWE that occurred in seven of the 13 samples (MAF > 99%, Figure 3a). These deviations reflected genomewide patterns, rather than the impact of a few extreme loci or genomic regions (e.g., inversions) as indicated by the consistency of the 95% CI along the autosomes for the samples exhibiting Fis deviations (Figure 3b). Unlike the two *A. gambiae s.s*. populations that appear to be in HWE, two *A. coluzzii* samples presented significant excesses of heterozygotes (Kolimana 2009 and most noticeably the May 2009 from Thierola), while seven other samples presented heterozygote deficiency (Figure 3). Notably, both *A. coluzzii* samples in agreement with HWE were collected in the mid‐RS (August of 2008 and 2010), whereas the highest and lowest Fis were both observed shortly after the first rain (May 2009 and June 2010, 3–8 and 2–18 days, after the first rain, respectively). While heterozygote deficits may represent Wahlund's effect (the pooling of two isolated reproductive units) in small populations, it is difficult to reconcile this explanation with deficits in the very large “perennial” population of Niono (Discussion).

### 3.3 Genetic distance among populations and samples

**Figure 4:**
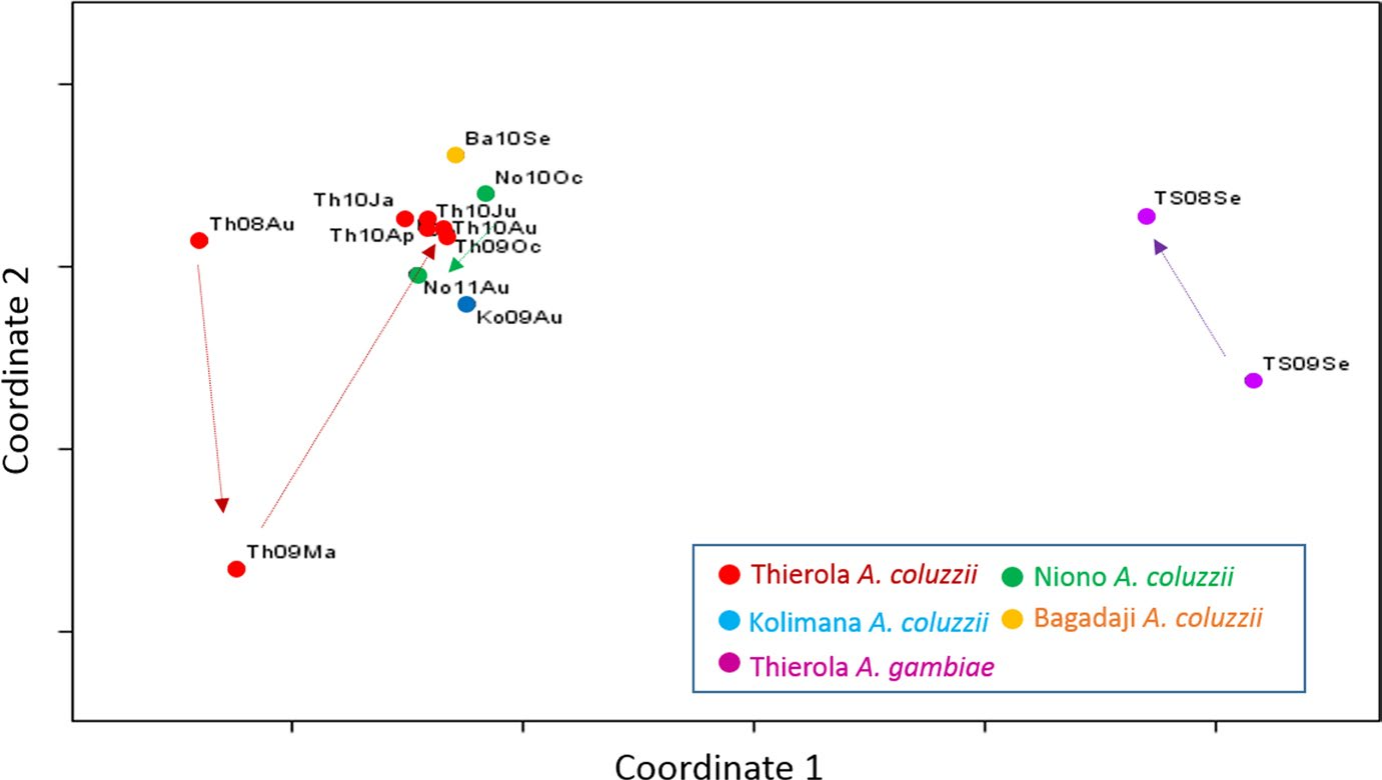
Genetic (Euclidian) distance between sample pairs portrayed by multidimensional scaling. Arrows denote sequence of sample collection (from the past to recent). The population sample's label follows Table 2

Overall genetic distances (Euclidian) between samples were computed across all 738 loci and used to depict the similarities among *A. coluzzii* and *A. gambiae* populations via multidimensional scaling (MDS, Figure 4) and a dendrogram (based on the UPGMA, Fig. S4). As expected, the deepest division separated *A. coluzzii* from *A. gambiae* (Figure 4). Accordingly, the first coordinate of the MDS separated the species and to a lesser extent the two *A. coluzzii* samples preceding September 2009 from later samples from Thierola. The second coordinate separated the Thierola sample of *A. coluzzii* from May 2009 from all other *A. coluzzii* samples (Figure 4). Among *A. coluzzii* samples, the MDS and the dendrogram revealed tight grouping of five samples from Thierola spanning the end of the RS through the DS and the mid subsequent RS (October 2009 to August 2010), surrounded by samples from Niono (October 2011 and August 2010) Bagadaji (September 2010) and Kolimana (August 2009, Figures 4 and S4). Considerable distances separated this grouping from earlier Thierola samples: August‐2008 and especially May‐2009 (Figures 4 and S4). This separation reflects a large change in allele frequencies in Thierola that occurred during the first year (August 2008 to September 2009) but minimal change subsequently. Considerable distance between the two *A. gambiae* samples from Thierola suggests that they too underwent substantial change in allele frequency, whereas the temporal change separating the Niono samples from October 2010 and August 2011 was rather modest. This pattern remained stable after excluding chromosomal regions such as chromosome 2, 2La, 2Rbc, 2R, although the separation of the Thierolan August 2008 sample from the main cluster was supported mostly by excluding 2La and 2Rbc (not shown).

**Figure 5:**
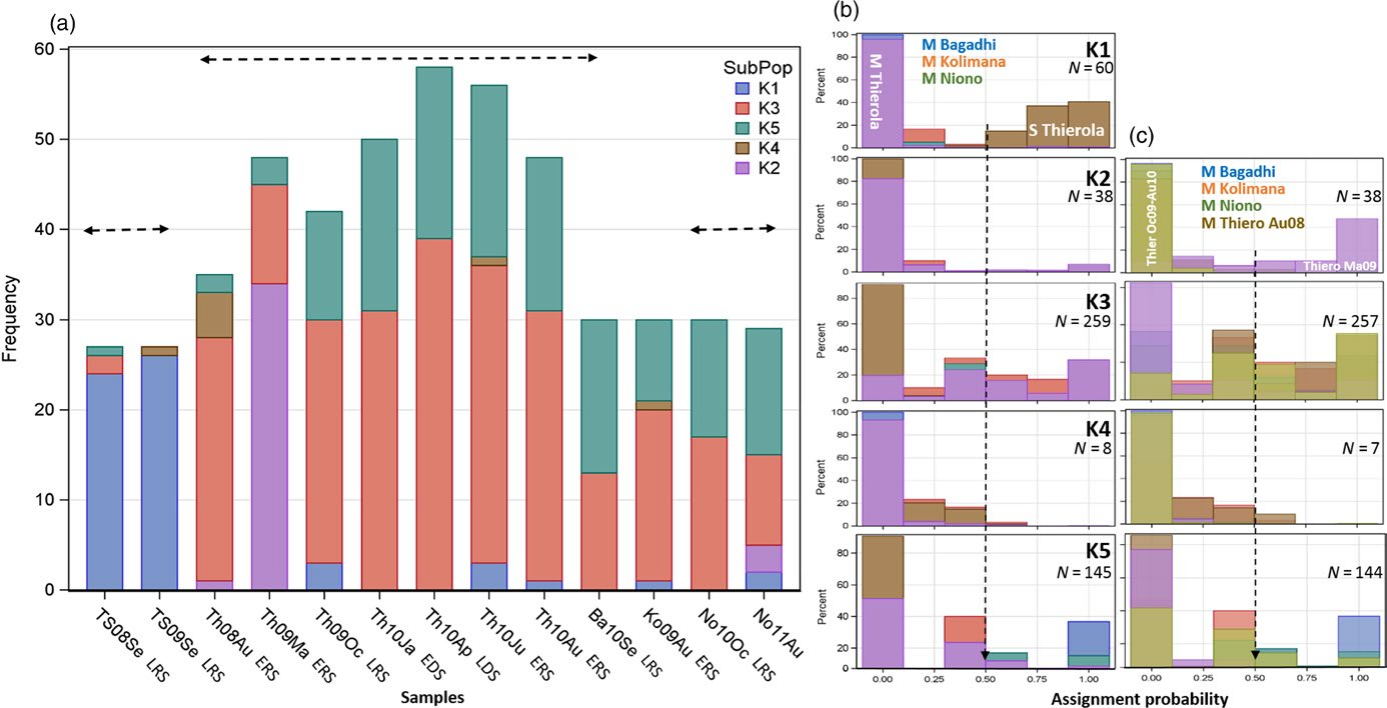
Division of samples (M and S denote *Anopheles coluzzii* and *Anopheles gambiae*, respectively) among the clusters inferred by Structure. The total sample was subdivided into five clusters (*K* = 5, Fig. S5). (a) Composition of each sample with respect to the five clusters identified by Structure. Horizontal lines indicate series of temporal samples taken from the same species and location. The seasonal acronyms under the *x*‐axis include “RS” and “DS” to denote rainy and dry seasons, preceded by “E” or “L” to denote the early and late parts of each season, respectively. (b) Composition of each cluster inferred by Structure with respect to all of the samples grouped by species and location based on the assignment probability (*x*‐axis). Vertical broken line points to 50% assignment, above which individuals may be assigned to that cluster. (c) Composition of each cluster inferred by Structure with respect to the samples of *A. coluzzii* by location. Note that the samples of Th08Au and Th09Ma are each separated from the remaining of the *A. coluzzii* samples of Thierola. Note that colours in panels a, b and c correspond to different keys

Assignment of individuals to subpopulations based solely on their multilocus genotype (irrespective of species) suggested a hierarchical clustering solution with optima at two and five groups (*K* = 2 and *K* = 5, Fig. S5). The sharp division between species (*K*1) and to a similar degree the separation of *A. coluzzii* into a subpopulation (*K*2) predominating in Thierola during late May 2009 (Th09Ma) were followed by more subtle subdivisions identifying three additional subpopulations (Figure 5). Only 12 specimens had maximal assignment probability to one of the five subpopulations below 0.5 and 325 mosquitoes had maximal assignment probability above 60%, indicating robust assignment. Very similar results and grouping of individual mosquitoes were produced by excluding the second chromosome (not shown), thus indicating that the clustering was not driven by variation within inversions, unless inversions were linked to genotypes in the 3^rd^ chromosome.

The *A. coluzzii* sample collected in May 2009 from Thierola (Th09Ma) consisted of a large fraction of cluster #2, which was absent elsewhere or at low frequency (earlier in Thierola and later in Niono; Figure 5b,c). Clusters #3 and #5 predominated throughout *A. coluzzii* samples, with the latter being relatively rare in the earlier samples from Thierola. Cluster composition was consistent for *A. coluzzii* samples from October 2009 to August 2010 (exact test based on likelihood ratio χ^2^_*df* = 8_ = 3.8, *p* > .9, Figures 5a,c), whereas inclusion of one or both of the earlier samples (Th08Au and Th09Ma), which also differed from each other (exact test based on likelihood ratio, χ^2^_*df* = 3_ = 42, *p* < .0001), revealed heterogeneity in cluster composition (exact test based on likelihood ratio χ^2^ > 30, *p* < .0002). Clusters #1, #3 and #5 exhibited continuity throughout temporal samples of corresponding species and populations, unlike subpopulations 2 and 4, which were detected during the early RS (May–August), but not in other times. The clusters probably do not represent sympatrically isolated reproductive units, and so the heterogeneous composition of Sahelian *A. coluzzii* suggests admixture between a local and distant populations, although a stable existence of co‐adapted genes by selection cannot be ruled out altogether. The relatively stable composition of clusters #3 and #5 and the erratic appearance of #2 and #4 are not consistent with seasonal selection. Within each cluster, departures from HWE have mostly persisted (Fig. S6), which does not support a stable admixture of semi‐isolated populations.

### 3.4 Testing “reproductive arrest” during the DS

A key prediction of aestivation is reproductive arrest during the DS. This arrest applies to the mosquitoes that initiate aestivation at the end of the RS until they resume reproduction after the first rain (Adamou et al., 2011; Dao et al., 2014; Lehmann et al., 2010). DS reproductive arrest implies minimal genetic drift between samples collected during that period, whereas the migration hypothesis predicts high drift because new migrants arrive from distant population(s) that continuously reproduce, albeit in smaller numbers during the DS. Therefore, under aestivation, the variance in allele frequency between two samples of the same generation should solely reflect sampling variation (assuming negligible selection). On the other hand, without aestivation, that is, continuous reproduction, these samples were taken several generations apart and an additional component of variance, given by their Ne, would be discernible. Accordingly, if the total variance in allele frequency (*F* ^*T*^) is larger than the experimenter's sampling variance (*V* ^*T*^), genetic drift is evident, consistent with reproduction (see Section 2). Tests of genetic drift (reproduction) were conducted between all pairwise samples in Thierola (by species) and Niono (Table 3). For *A. coluzzii* in Thierola, all but one sample pair with one of the samples preceding October 2009 exhibited *F* ^*T*^ > *V* ^*T*^, whereas *F* ^*T*^ of all other sample pairs were smaller or equal to *V* ^*T*^ (Table 3). Importantly, these results showed no evidence of genetic drift across the five samples spanning the DS (October 2009 to June 2010) and a measurable drift between August 2008 and May 2009 (and between these time points and all subsequent samples). Consistent with aestivation, there is no evidence of reproduction (evident as measurable drift) during the DS up to the week after the first rain (June), while there was evidence for reproduction for samples spanning the (2008 and 2009) rainy seasons. However, no evidence of drift was found in the early 2010 RS (June to August) contrary to this prediction. Despite year‐round reproduction, the pair of samples of *A. coluzzii* from Niono did not exhibit evidence of drift, consistent with a very large Ne. Contrary to the long‐distance migration prediction, the samples of *A. gambiae* in Thierola spanning almost a year showed no evidence of drift (Table 3), possibly reflecting smaller sample size of this species (Table 2), thus reducing the power for detection of *F* ^*T*^ > *V* ^*T*^.

**Table 3:**
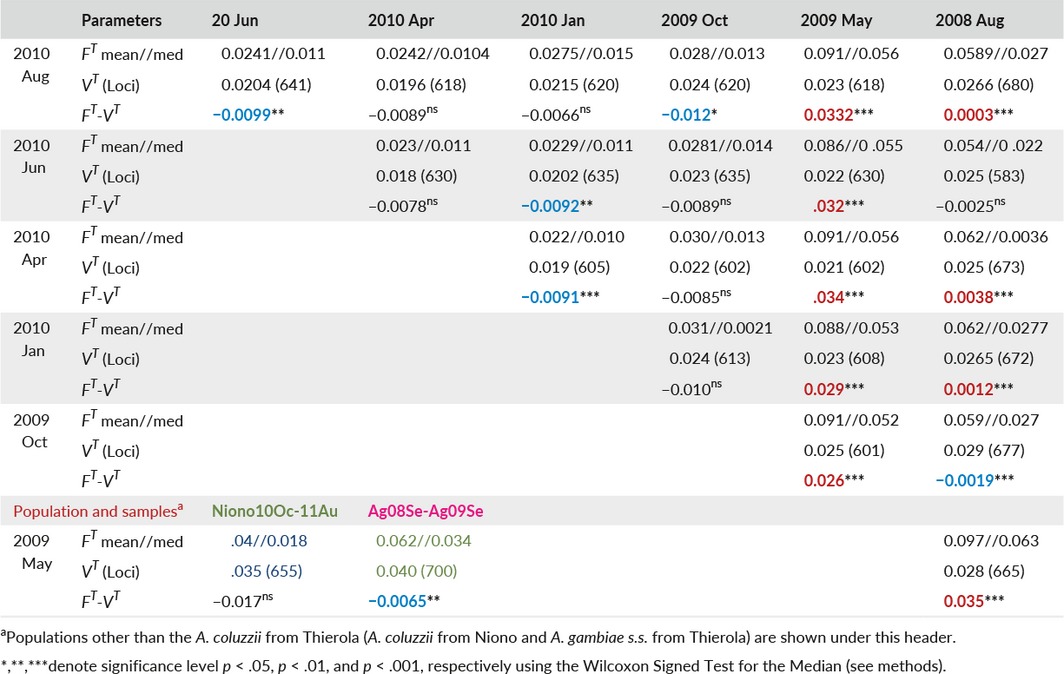
Temporal variation in allele frequency (*F* ^*T*^) between samples of *Anopheles coluzzii* (Thierola and Niono) and *Anopheles gambiae* (Thierola) in relation to experimental sampling variation (*V* ^*T*^) as a test of reproduction between time points, evident as genetic drift. Red highlighting indicates significantly higher total variance than sampling variance (measurable drift) and blue significantly higher sampling variance (no drift). Sample pairs of *A. coluzzii* from Thierola are shown above the diagonal and are listed according to the dates indicated by the columns and rows, whereas the sample pairs from Niono (green) and *A. gambiae* from Thierola (magenta) are shown below diagonal and their sampling dates listed

**Figure 6:**
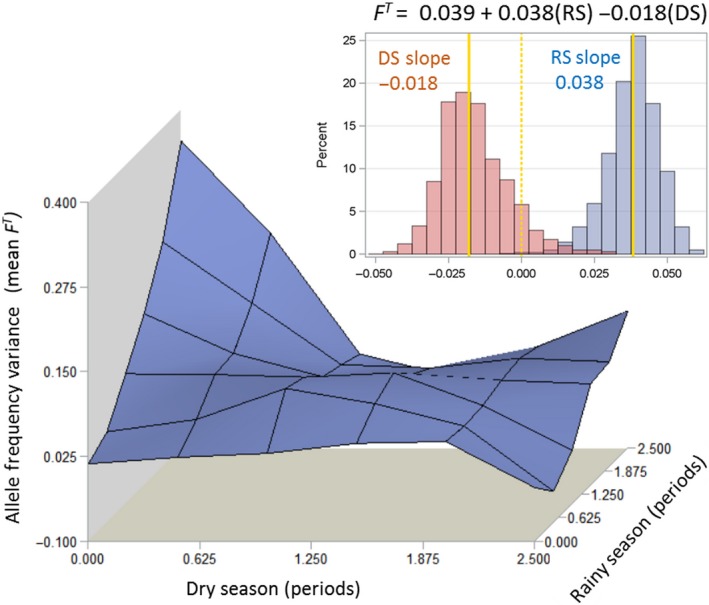
The effects of periods of dry season (*x*‐axis) and rainy season (*y*‐axis) on the mean variance in allele frequency, *F^T^*, (*z*‐axis) across loci in all pairwise sample combinations in Thierola (*n* = 21 comparisons, Table 3. See text for details). Response surface was generated using bivariate interpolation (Proc g3Grid in SAS). Inset: Confidence intervals of the regression coefficients (values marked by the solid yellow lines) measuring the change in the variance in allele frequency versus time for the DS and RS produced by bootstrapping (over the sample pairs, 1,000 pseudoreplicates)

To evaluate the overall season‐specific effects of DS and RS on drift in *A. coluzzii*, we regressed for each sample pair, their mean *F* ^*T*^ (across all loci) over the duration of RS and DS that separated them (Figure 6). Bootstrapping (1000 pseudoreplicates), used to determine the 95% CI around the season coefficients, revealed that the RS slope was positive (0.038, 95% CI = 0.018–0.054), consistent with occurrence of time‐dependent drift, whereas the DS slope was not significantly greater than zero (−0.018, 95% CI = −0.035 – 0.0124, Figure 6). Whether the regression excluded or included the intercept, the significance of the RS and DS effects, as determined by bootstrapping were the same (not shown).

## 4 DISCUSSION

Using genetic data, we aimed to determine whether in the Sahel, the early RS population of *A. coluzzii* descended from the preceding late RS generation at the same locality, consistent with aestivation, or from migrants from distant locations. These hypotheses were proposed for different malaria mosquito species based on recent studies of population dynamics, mark–release–recapture, semi‐field cage experiments and seasonal changes in behaviour, physiology and morphology (Adamou et al., 2011; Arcaz et al., 2016; Dao et al., 2014; Huestis & Lehmann, 2014; Huestis et al., 2012; Lehmann et al., 2010, 2014; Mamai, Mouline, et al., 2016; Mamai, Simard, et al., 2016; Yaro et al., 2012). However, the inability to find direct evidence, such as mosquitoes in their shelters during the DS, has prevented a definitive resolution of this problem. Using 738 SNPs genotyped in eleven *A. coluzzii* samples, including seven temporal samples spanning two years in one location, and two *A. gambiae* samples from the same location, we evaluated predictions (see Table 1) based on these two alternative hypotheses. Rather than providing a solid fit to one or the other hypotheses, the results present a mosaic of good and poor fit with respect to both sets of predictions, necessitating reassessment of underlying scenarios and consideration of additional ones.

The central predictions (1.1–1.4, Table 1) based on aestivation as distinct from those based on long‐distance migration (2.1–2.4) were the following. Owing to their local origin, minimal genetic distance was predicted among samples taken from the same locality from the beginning of the DS and until immediately after the first rain of the following RS (~7 months later), whereas larger genetic distance was expected between samples spanning the RS (see prediction 1.3). Moreover, owing to reproductive arrest during the DS and the survival of just hundreds of mosquitoes, no genetic drift was expected between samples taken at the same Sahelian site during the DS and immediately after the first rain, despite containing the period with the lowest population density (prediction 1.4); yet, measurable drift is predicted over the RS. The results revealed that the genetic distances among the four samples of *A. coluzzii* from the end of the RS and until the ensuing RS (October 2009 to June 2010) were small and these samples were clustered together and away from those separated by a RS (August 2008 and May 2009, Figure 4), according to prediction 1.3 (Table 1) and contrary to 2.3, above. Likewise, assignment of individuals to clusters based solely on their multilocus genotype using Structure revealed a similar composition of samples taken from the end of the RS and until after the first rain of the following RS, but a distinct composition for samples taken across the RS (Figure 5). Additionally, genetic drift was detected between samples spanning the RS but not between samples spanning solely a DS (Table 3). Further, the temporal variance in allele frequency increased with time over the RS but not over the DS (Figure 6), in accordance with prediction 1.4 but contrary to 2.4.

Although the results above support the main predictions of aestivation, other results did not fit well: (i) The genetic distances separating the two earliest *A. coluzzii* samples from Thierola from each other and from all other subsequent samples, although taken a year apart, were conspicuously large (Figure 4), contrary to the stable genetic constitution predicted by hundreds of aestivators that survive the DS (Adamou et al., 2011). The large difference in cluster composition of these samples in comparison with all other *A. coluzzii* samples (collected subsequently, Figure 5) corroborates this finding. In fact, the genetic distance between samples taken a year apart in 2008–2009 (though not in 2009–2010) was greater than between geographical samples taken 50–130 km away (Kolimana and Niono, Figures 4 and 5). These results support a bottleneck/extinction process followed by migration occurring in the first year (but not the second) in *A. coluzzii*. The within‐sample admixture indicated by Structure in *A. coluzzii* (Figure 5) further supports long‐distance migration. Such sympatric subpopulations include clusters #2 and #4 that appeared and disappeared over a period of a few months, while #3 and #5 exhibited continuity over time in all the Sahelian populations. These subpopulations are unlikely to represent sympatric isolated reproductive units, especially because the deviations from HWE did not dissipate when calculated separately within the clusters (Figures 3 and S6). The heterogeneity of Sahelian *A. coluzzii* consisting of four subpopulations suggests admixture between a local and distant population(s), probably via long‐distance migration, which would take several generations to dissipate across loci.

Additionally, (ii) contrary to prediction (1.1), within‐population diversity in *A. coluzzii* varied markedly over time in Thierola (Figure 2), but the changes did not follow simple seasonal expectations such as low diversity in the DS and high diversity in the RS. Seasonal variation between temporal samples was also documented in Sahelian *A. arabiensis* from Senegal and Cameroon (Simard et al., 2000; Wondji, Simard, et al., 2005). Typically, a decline in genetic diversity indicates a past reduction in Ne, whereas an increase over a few months indicates migration. The earliest and consecutive samples of August 2008 and May 2009 have produced the highest and lowest diversity among all *A. coluzzii* samples, respectively (Figure 2). This dramatic decline is consistent with insecticide‐induced bottleneck, following the mass distribution of insecticide‐treated nets (ITNs) that started in March–June 2008 (Plan 2009). Additional support for the insecticide‐induced bottleneck is provided by the significant increase in the *Vgsc*‐1014F resistant mutation from 13% to 36% between these time points (Fig. S2), and by the >6‐fold lower peak density of *A. coluzzii* during that RS (Dao et al., 2014). This insecticide‐induced bottleneck could also explain the conspicuously large genetic distance between these samples and all others (Figures 4, 5 and Table 3). Notably, the subsequent rise in genetic diversity occurred after the population surge following the first rains, indicating migration during the RS and not during the DS or with the first rain; based on the similarity of the August 2010 to previous samples in the preceding DS, long‐distance migration occurred in the late RS (August‐October).

The departures from HWE (Figure 3) are an unusual aspect of the current study. Migrant mated females arriving at the late RS (August‐October), carrying different alleles, may generate heterozygote deficits (Wahlund effect, positive Fis; Figures 4 and S4). As reproduction ceases in the Sahel due to the absence of surface waters across vast areas such as around Thierola, these heterozygote deficits would persist until reproduction resumes a few weeks after the first rain (e.g., Th10Au Figure 3). These deviations, although mild (0 < Fis < 0.1), suggest massive migration, which presumably follows the prevailing winds, and accordingly arrive from the south. As nearby *A. coluzzii* populations were genetically similar, such migration must arrive from a distant area (>150 km). The heterozygote deficits observed in Niono may also attest for admixture with long‐distance migrants and/or with Sahelian populations that surround the narrow (~5 km wide) irrigated area.

The exceptional value of heterozygote excess manifested by the 2009 *A. coluzzii* sample of Thierola from May and to a lower degree by the Kolimana sample from August (Figure 3) are difficult to explain and similar values have not been observed previously in *A. gambiae* species (Lehmann et al., 1998, 2003; Slotman et al., 2007; Wondji, Frederic, et al. 2005). The heterozygote excess might reflect an aftermath of the insecticide treatment. Accordingly, the local Sahelian‐adapted population was reduced by ITNs, whereas many of the migrants carried mutations conferring resistance to the insecticides, such as the *Vgsc*‐1014F. During the Sahelian DS, those genotypes were selected against, resulting in the preferential survival of heterozygotes that persisted until the first rains of 2009. Possibly such DS‐related constraints cap the frequency of the *Vgsc*‐1014F (and possibly other mutations) in Sahelian *A. coluzzii* (Fig. S2). Under a severe bottleneck, different alleles are fixed in females and males and their offspring would present heterozygote excess (Balloux, 2004), but this seems unlikely given the observed density (Lehmann et al., 2010). Finally, it is possible that aestivation involves a small fraction of the population, consisting of individuals carrying a combination of alleles from several polymorphic loci. These genotypes are favoured by the harsh Sahelian DS, yet are selected against during the RS, possibly generating subpopulations with a degree of linkage across the genome that produces the subpopulations identified by Structure as well as the departures from HWE as recently been demonstrated in overwintering *Drosophila melanogaster* in the temperate zone (Bergland, Behrman, O'Brien, Schmidt, & Petrov, 2014).

Having only two samples of *A. gambiae* precludes a comprehensive analysis of their fit to key predictions based on migration as previously proposed (Adamou et al., 2011; Dao et al., 2014). Yet, the observed patterns revealed large variation in within‐population diversity, possibly consistent with prediction 2.1. In accordance with 2.3, considerable genetic distance separated the samples, possibly reflecting a founder effect, but contrary to 2.4, genetic drift was not detected between the two samples, providing no evidence for continuous reproduction (Table 3), possibly due to the lower power resulting from smaller samples.

In conclusion, our results reveal strong but imperfect agreement with predictions based solely on aestivation in *A. coluzzii*. Altogether, the observed patterns in this species support aestivation accompanied by long‐distance migration in the late RS. Such migration does not address the problem of persistence throughout the DS, but could be important for persistence through the unpredictable dry spells during the RS, which are rather common in the Sahel (Hastenrath & Polzin, 2011, 2014; Salack, Giannini, Diakhate, Gaye, & Muller, 2014; Tarhule et al., 2015). Without migration, every Sahelian population that survives the DS via aestivation would become extinct because of RS dry spell(s) at this or another year. Aerial sampling of mosquitoes at 100–250 m above ground yielded *A. coluzzii* mosquitoes from August to October (A. Dao, A.S. Yaro, D.L. Huestis, M. Diallo, Z. Sonogo, S. Djibril & T. Lehmann, unpublished data) provides additional compelling evidence for the involvement of long‐distance immigrants. Therefore, we propose that in *A. coluzzii* (unlike *A. gambiae*), long‐distance migration is a strategy to contend with dry spells during the Sahelian RS. Our results also indicate an ephemeral bottleneck that accompanied the mass distribution of ITNs in that year (2008) resulting in high variance in within‐ and between‐population diversity. This bottleneck disrupted the typical dynamics of demographic and thus genetic parameters during the first year of our study. A follow‐up study with a longer time series and a greater coverage of the genome would help narrow the uncertainty about the origin of the early RS *A. coluzzii* in the Sahel and also evaluate the possibility of seasonal selection.

## CONFLICT OF INTEREST

All authors declare no competing financial interests with respect to any aspect of this article.

## DATA ARCHIVING STATEMENT

SNPs data used in the analyses here are available through the Dryad Digital Repository: https://doi.org/10.5061/dryad.t5k6f.
