# Supplementary material for: Tracing the origin of the early wet‐season Anopheles coluzzii in the Sahel
Source: Evol Appl. 2017 May 26;10(7):704–17. doi: 10.1111/eva.12486 (PMC5511357; doi:10.1111/eva.12486)
Supplement: Supplementary file 1 [file EVA-10-704-s001.docx]

**Tracing the origin of the early wet-season *Anopheles coluzzii* in the Sahel**

**Lehmann, Tovi**^a^**;Weetman, David**^b^**; Huestis Diana, L.**^a^**, Yaro,. Alpha S.**^c^**; Kassogue**^c^ **Yaya, Diallo, Moussa**^c^**; Donnelly, Martin, J.**^b^ **and Dao, Adama**^c^

^a^ Laboratory of Malaria and Vector Research, NIAID, NIH. Rockville, MD, USA

^b^ Department of Vector Biology, Liverpool School of Tropical Medicine, Liverpool, UK,

^c^ Malaria Research and Training Center (MRTC)/Faculty of Medicine, Pharmacy and Odonto-stomatology, Bamako, Mali


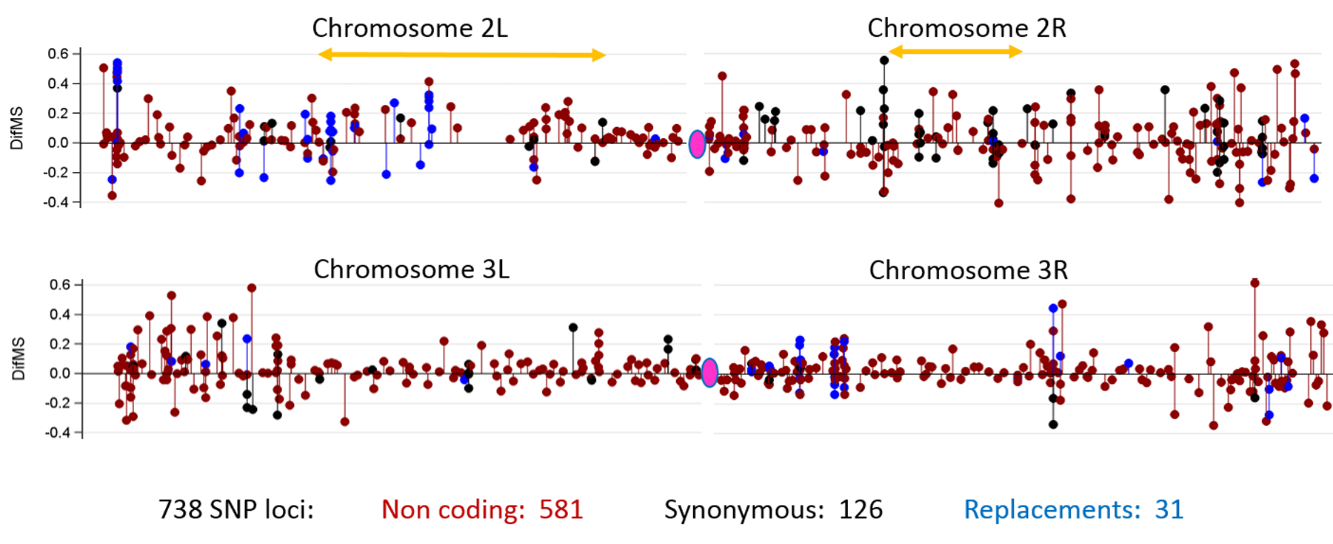


**Figure S1.** Distributions of SNPs included in the analysis along the chromosomes showing species difference (DifMS = *A. coluzzii – A. gambiae s.s*.) in allele frequencies by SNP type (NCD, synonymous, replacement). Position of inversions 2La and 2Rbc are schematically shown by horizontal arrows and the centromeres by the red ellipses.

**
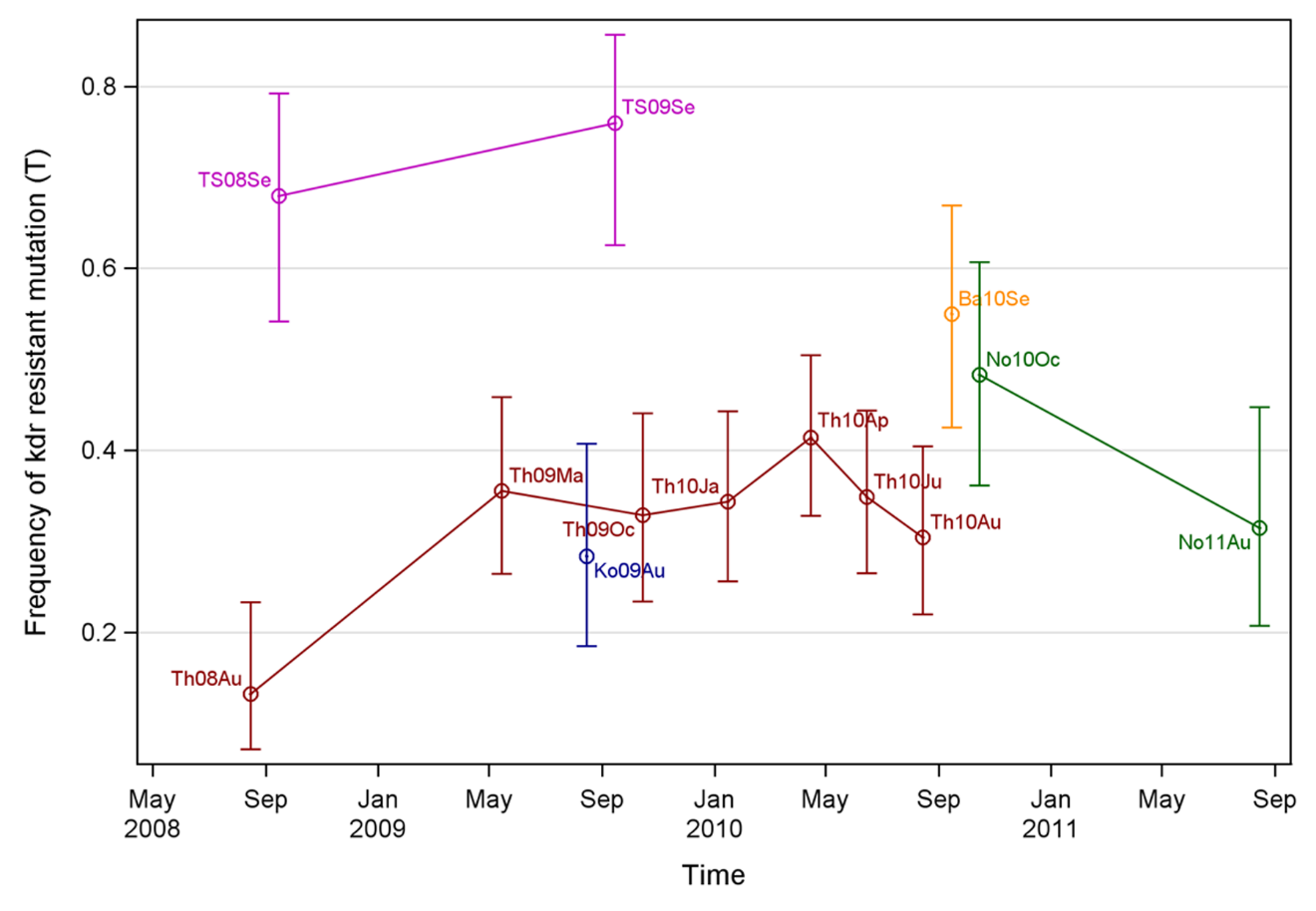
**

**Figure S2.** Change in frequency of *Vgsc*-1014F resistant-allele mutation (A🡺T) over time in the samples.

**
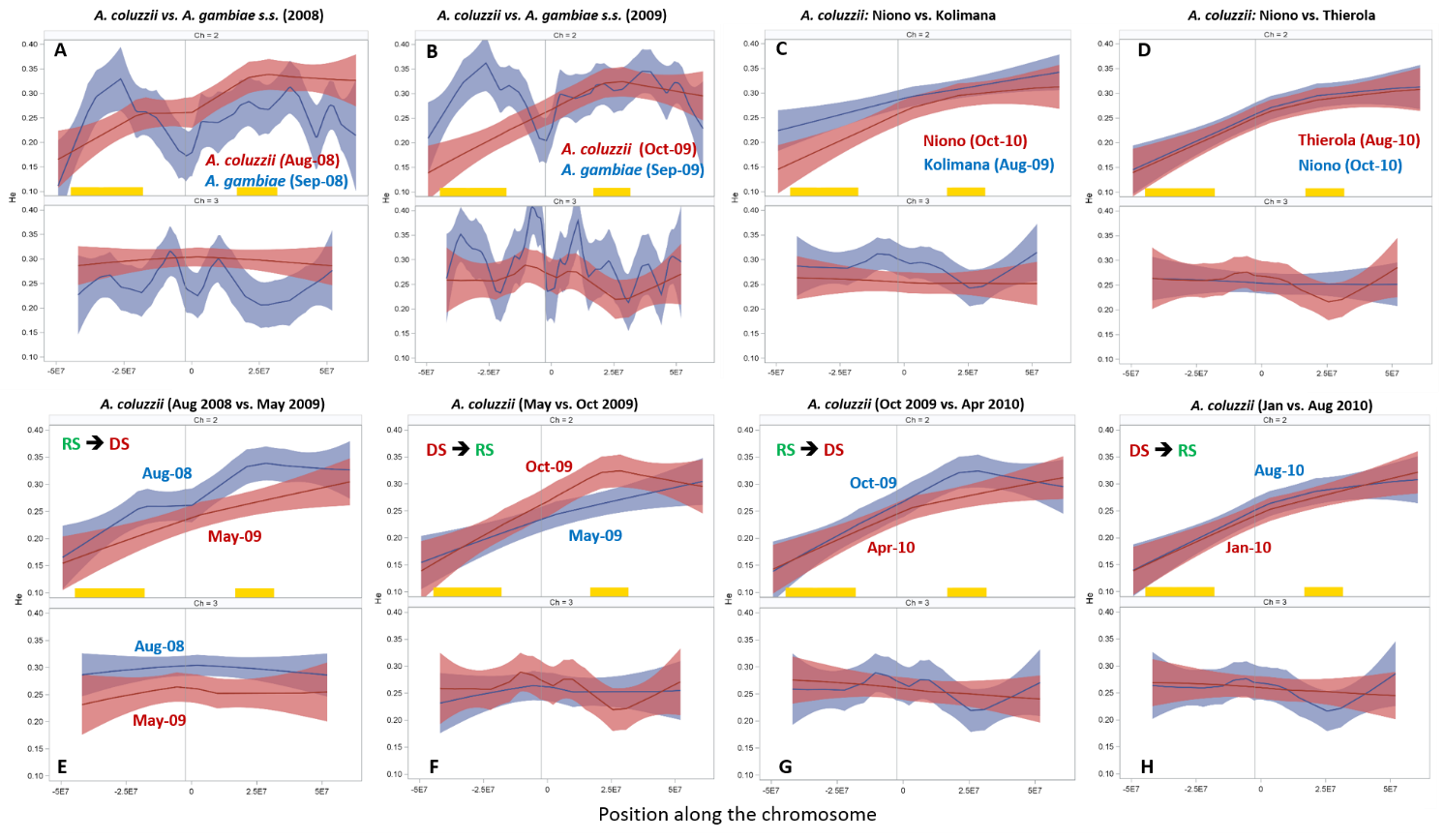
**

**Figure S3.** Within-sample diversity (*He*) along the autosomes (second and third chromosomes in the top and bottom panels, respectively) in sample pairs. Species comparisons of contemporaneous *A. coluzzii* and *A. gambiae* from Thierola (A: 2008 and B: 2009, top left), locality differences between *A. coluzzii* from Kolimana vs. Niono (C) and Thierola and Niono (D, top right), and cross seasonal variation between successive time points in Thierola shown in the bottom panels (E-H). Nonparametric (loess) regression functions and 95% CI are denoted by color for each sample (note: colors alternate across plots). The position along the chromosome (x axis) shows the centromere as zero and negative values represent the left arm. Solid yellow bars denote positions of inversion 2La and 2Rbc on the second chromosome and vertical line denotes position of the *Vgsc*-1014F mutation on chromosome two.

**
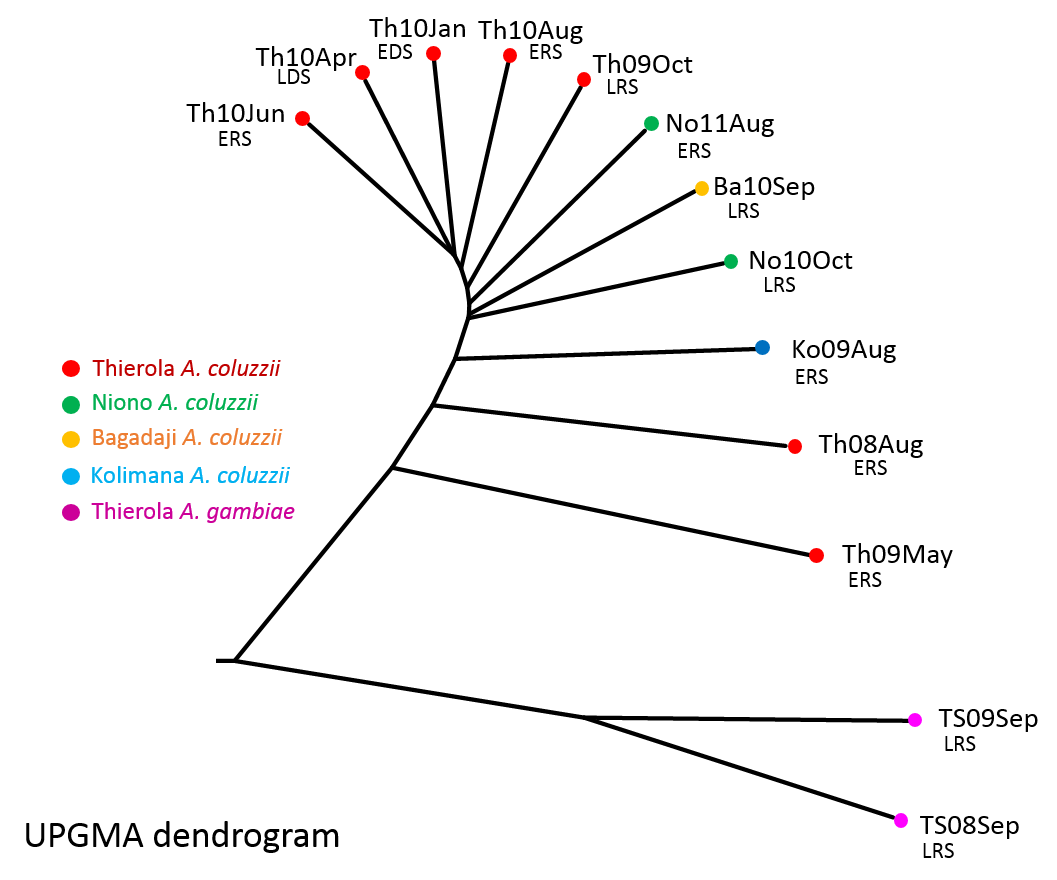
**

**Figure S4.** Dendogram showing genetic distance between samples based on UPGMA on the Euclidean distance in allele frequency across loci. Genetic (Euclidian) distance between sample pairs portrayed by multi-dimensional scaling (MDS). Color denote populations (year and month of samples are provided in the sample name). The season acronym includes ‘RS’ and ‘DS’ to denote rainy and dry seasons, preceded by ‘E’ or ‘L’ to denote the early and late parts of each season, respectively.

**
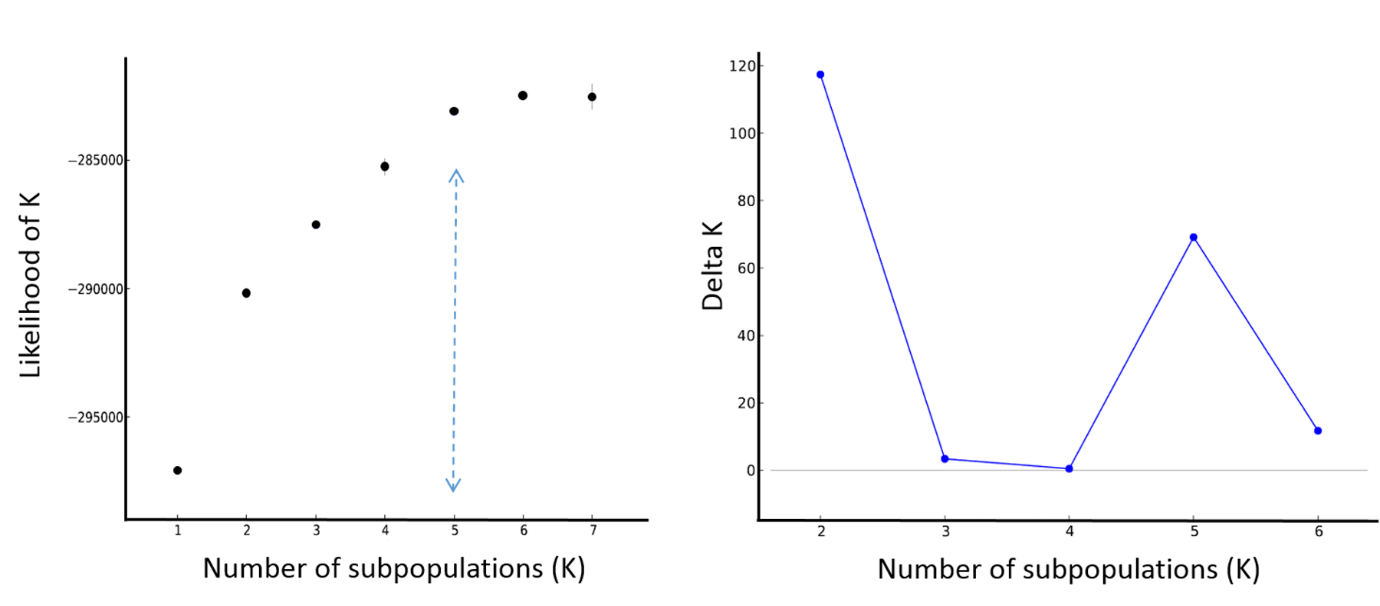
**

**Figure S5.** The optimal value of the number of subpopulations (K) in the total sample (N=510) based on Structure likelihood parameters. (A) shows the mean likelihood (±SD) approaching maximum at K=5 or 6 and (B) the delta K parameter (see Materials and Methods for details), suggesting optimal K values of 2 and 5.

**
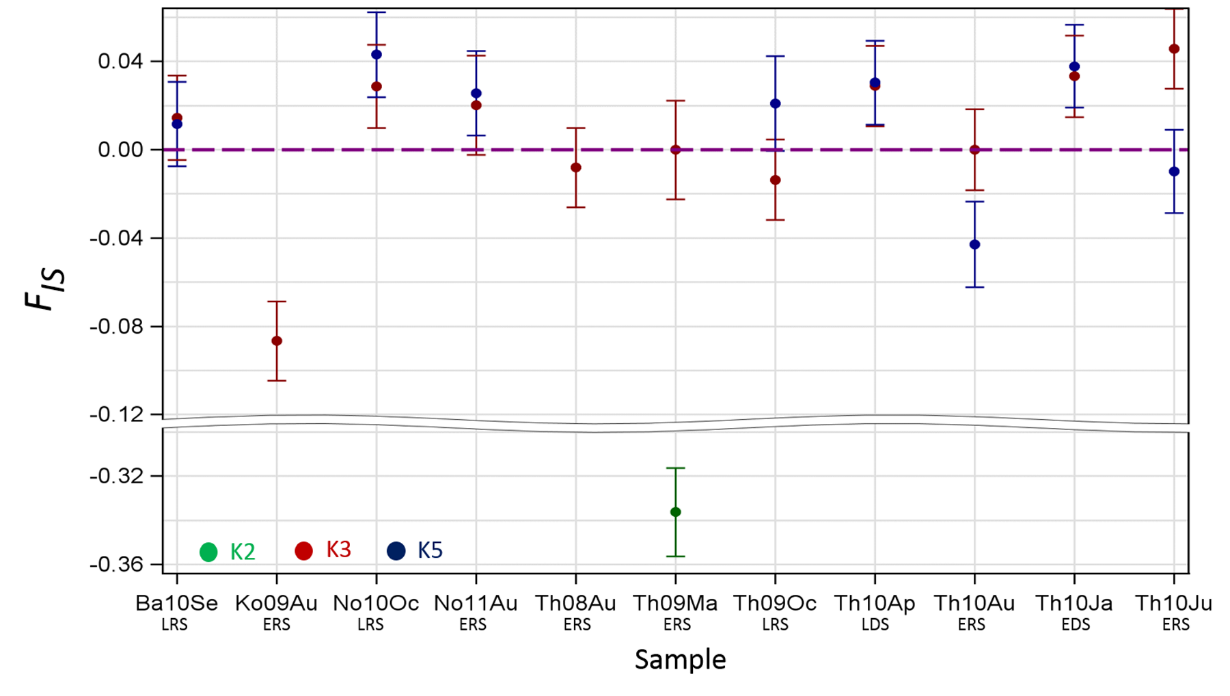
**

**Figure S6.** Departures from HWE measured by least squares means of locus-specific Fis in each subpopulation identified by Structure (K2, K3, and K5, see text and Fig. 5) of each A. coluzzii sample. Only loci with common allele frequency <99% and total sample size per subpopulation >9.
